# Fulminant necrotizing fasciitis following the use of herbal concoction: a case report

**DOI:** 10.1186/1752-1947-4-326

**Published:** 2010-10-19

**Authors:** Ismaila A Adigun, Abdulrasheed A Nasir, Adebiyi B Aderibigbe

**Affiliations:** 1 Division of Plastic and Reconstruction Surgery, Department of Surgery, University of Ilorin Teaching Hospital, PMB 1459, Ilorin, Nigeria; 2 Department of Surgery, University of Ilorin Teaching Hospital, PMB 1459, Ilorin, Nigeria

## Abstract

**Introduction:**

Necrotizing fasciitis is a rare and life-threatening rapidly progressive soft tissue infection. A fulminant case could involve muscle and bone. Necrotizing fasciitis after corticosteroid therapy and intramuscular injection of non-steroidal anti-inflammatory drugs has been reported. We present a case of fulminant necrotizing fasciitis occurring in a patient who used a herbal concoction to treat a chronic leg ulcer.

**Case presentation:**

A 20-year-old Ibo woman from Nigeria presented with a three-year history of recurrent chronic ulcer of the right leg. She started applying a herbal concoction to dress the wound two weeks prior to presentation. This resulted in rapidly progressive soft tissue necrosis that spread from the soft tissue to the bone, despite aggressive emergency debridement. As a result she underwent above-knee amputation.

**Conclusion:**

The herbal concoction used is toxic, and can initiate and exacerbate necrotizing fasciitis. Its use for wound dressing should be discouraged.

## Introduction

Necrotizing fasciitis (NF) is a rare but fatal rapidly progressive soft tissue infection, which is characterized by widespread necrosis of the superficial fascia and the subcutaneous fat. NF spreads along the fascia plane usually sparing surface skin and the muscle but fulminating cases can affect the muscle. NF is associated with high mortality and long-term morbidity [1,2]. Early clinical suspicion of necrotizing fasciitis is crucial because patient survival is inversely related to the time between the onset of infection and the initiation of appropriate therapy [1]. Fulminating necrotizing fasciitis after corticosteroid therapy [3] and intramuscular injection of nonsteroidal anti-inflammatory have been reported [2]. Fulminant necrotizing fasciitis from an herbal concoction has not been reported in English-language medical literature. We present a case of fulminating NF that occurred after the use of an herbal concoction.

## Case presentation

**Figure 1:**
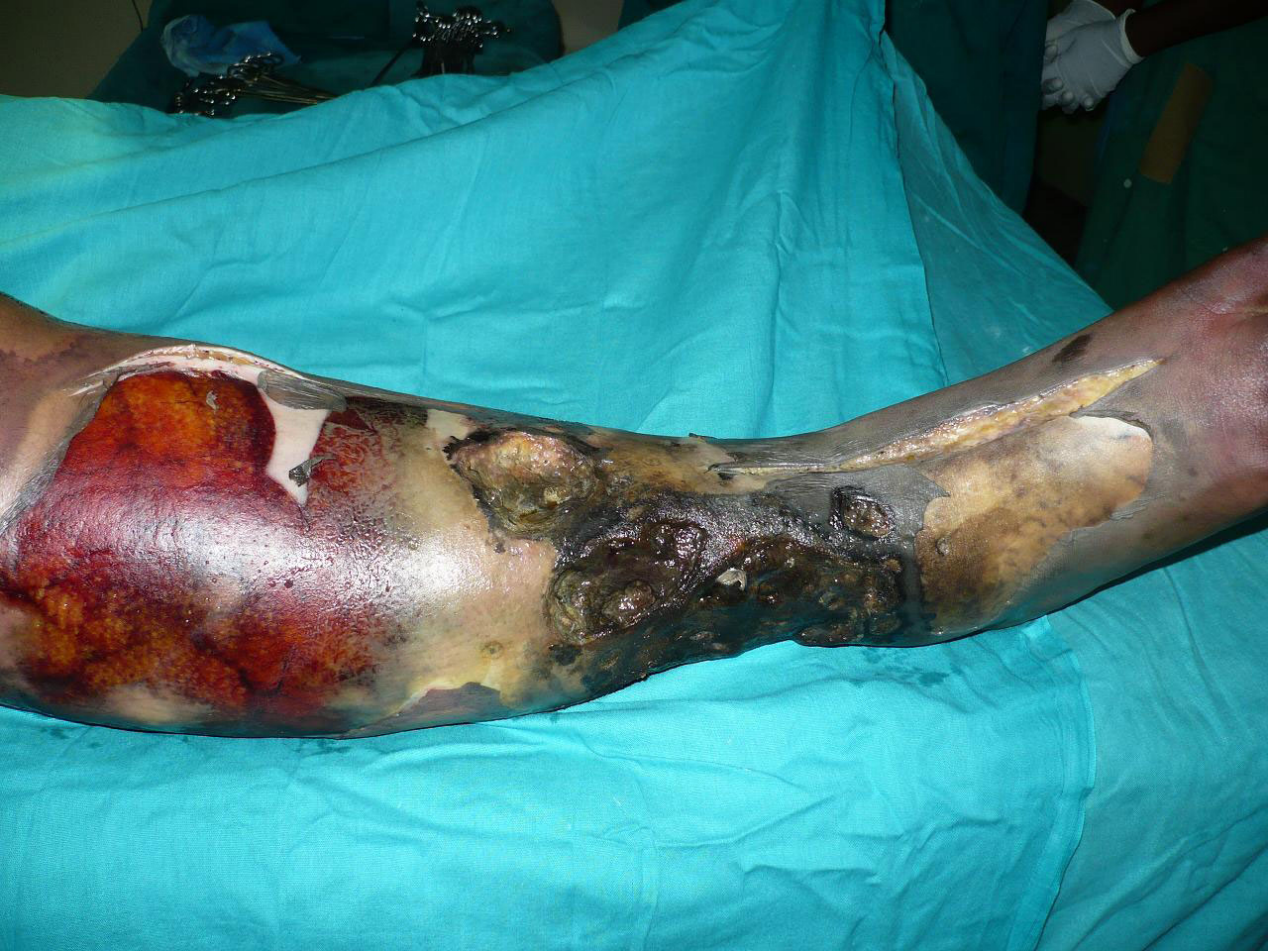
**Right leg showing progressive skin necrosis up to the upper third of the leg, with incision of initial fasciotomy on the dorsum of the foot and distal leg**. This was on the third day after admission.

**Figure 2:**
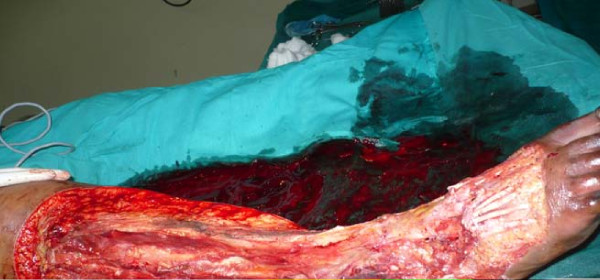
**Right leg immediately after initial radical debridement showing well vascularised soft tissue on the fourth day after admission**.

**Figure 3:**
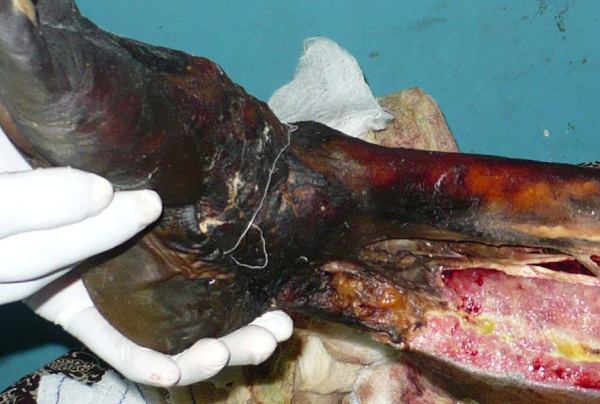
**Progressive right leg tissue necrosis involving the tibia and fibula a week after initial debridement**.

A 20-year-old Ibo undergraduate woman presented with a three-year history of recurrent chronic ulcer of the right leg. Upon presentation there was associated leg pain and progressive leg swelling of three days duration. The leg ulcer had started increasing progressively in size following the use of an herbal concoction the patient used to dress the wound two weeks prior to presentation. She had had two previous skin grafts. She, however, had not attended a follow-up appointment. Upon presentation she was a young, overweight woman, toxic and dehydrated. She was febrile with a temperature of 38.2°C, had tachypnea, and was jaundiced with bilateral pitting pedal edema which was worse on the right leg up to the thigh. She had a regular pulse of 132 beats per minute with blood pressure of 90/50 mmHg. There were multiple ulcers on the right leg. The floor of the ulcers contained slough with purulent discharges. The dorsalis pedis was not palpable on the right side. The packed cell volume was 20% and serum electrolytes were normal. She was resuscitated with 0.9% normal saline, intravenous antibiotic ceftriazole 1 g every twelve hours, metronidazole 500 mg every eight hours. She was transfused with three units of blood. Initial fasciotomy was performed with no significant improvement. She developed widespread skin necrosis and palpable crepitus on her right foot up to the upper third of her leg (Figure 1). She had emergency radical debridement of the anterior, lateral and part of the posterior compartments of the right leg on the fourth day following admission. Intraoperative findings were large intramuscular abscesses, and myonecrosis of the gastrocnemius and the tibialis anterior muscle, which were debrided (Figure 2). She was treated with a honey dressing and serial debridement. There was, however, progressive necrosis involving the tibia and fibula up to the level of her knee (Figure 3) and she became septic. She then underwent above-knee amputation (AKA) of the right extremity. Culture of the wound biopsy yielded mixed growth of *Klebseilla *and *Pseudomonas*. Histology of the debrided tissues showed fibromuscular and fibrofatty tissue with extensive necrosis and a focal collection of mononuclear inflammatory cells. She did well postoperatively and she is being prepared for prosthesis.

## Discussion

Necrotizing soft tissue infections are characterized by rapid progression of infection with soft tissue destruction and are associated with high long-term morbidity and mortality [1,2]. When muscle necrosis is involved, like that in the patient presented, the term myonecrosis is used [1]. NF can be initiated after surgical procedures, minor trauma, trivial scratches, or in the setting of a chronic wound, but may occur spontaneously or after minor injury in an otherwise healthy person [4,5]. In developing countries, an herbal concoction of unknown composition is used for various purposes but its use to treat wounds is uncommon. It is toxic and highly contaminating, and can initiate and exacerbate progression of soft tissue infection. Its use can lead to progressive necrosis in the patient. No case of amputation was recorded in the previous report by Adigun et al from the University of Ilorin Teaching Hospital [6]. The causative organisms are usually mixed and are toxin-producing, virulent bacteria, including *Streptococcus*, *Staphylococcus*, or a combination of Gram-negative bacilli and anaerobes [4,7]. Mixed growth of *Klebseilla *and *Pseudomonas *were cultured in our patient. Presentation is usually non-specific with minimal local manifestations. Symptoms are fever, leg pain and swelling, or early cutaneous signs including edema, erythema, local anesthesia and occasional crepitus. Despite the minimal local manifestations, the patients usually complain of severe pain. Pain out of proportion to the physical findings in a patient with systemic toxic signs should raise the clinical suspicion of necrotizing fasciitis [8]. In the late stage of the disease, the infection is disseminated through the relatively avascular fascia planes. It causes thrombosis of the affected blood vessels and devascularisation of the overlying skin. As organisms and toxins are released into the bloodstream, sepsis invariably develops [9]. The unexpected fulminant clinical course may point to diagnosis of myonecrosis as in this patient presented with progressive necrosis and sepsis despite initial fasciotomy and debridement. When necrotizing myositis is suspected, gross muscle necrosis can be confirmed by radiological imaging, such as computerized tomography and magnetic resonance imaging [1]. These technologies are not available in our center. The mainstay of treatment of all necrotizing soft-tissue infections is early radical debridement of all necrotic tissue. Fulminating cases may require amputation of the affected extremity. Clinical suspicion must prompt immediate surgical intervention with aggressive debridement and appropriate antibiotic therapy.

## Conclusion

The herbal concoction the patient used is a highly toxic contaminant that can lead to fulminating soft tissue infection. The use of this herbal concoction for wound care should be discouraged. An aggressive early surgical debridement is needed to prevent unnecessary amputation.

## Consent

Written informed consent was obtained from the patient for publication of this case report and any accompanying images. A copy of the written consent is available for review by the Editor-in-Chief of this journal.

## Competing interests

The authors declare that they have no competing interests.

## Authors' contributions

IAA operated on the patient and was a major contributor in writing the manuscript. AAN wrote the initial and final drafts, and did the revision of the manuscript and carried out the literature search. ABA wrote the case summary and was a major contributor in writing the manuscript. All authors read and approved the final manuscript.
